# Epidemiology of chronic multimorbidity and temporary migration in a rural South African community in health transition: A cross-sectional population-based analysis

**DOI:** 10.3389/fepid.2023.1054108

**Published:** 2023-03-21

**Authors:** Armstrong Dzomba, Carren Ginsburg, Chodziwadziwa W. Kabudula, Rachel R. Yorlets, Pedzisai Ndagurwa, Sadson Harawa, Mark N. Lurie, Stephen T. McGarvey, Stephen Tollman, Mark A. Collinson, Michael J. White, Francesc X. Gomez-Olive

**Affiliations:** ^1^ Medical Research Council/Wits Rural Public Health and Health Transitions Research Unit (Agincourt), School of Public Health, Faculty of Health Sciences, University of the Witwatersrand, Johannesburg, Gauteng Province, South Africa; ^2^ Department of Epidemiology, Brown University School of Public Health, Providence, RI, United States; ^3^ Population Studies and Training Center, Brown University, Providence, RI, United States; ^4^ South African Medical Research Council Vaccines and Infectious Diseases Analytics Research Unit, Faculty of Health Sciences, University of the Witwatersrand, Johannesburg, South Africa; ^5^ International Health Institute, Department of Epidemiology, School of Public Health, Brown University, Providence, RI, United States; ^6^ Department of Anthropology, Brown University, Providence, RI, United States; ^7^ Department of Science and Innovation/ Medical Research Council, South African Population Research Infrastructure Network, Durban, South Africa; ^8^ Department of Sociology, Brown University, Providence, RI, United States

**Keywords:** human migration, epidemics, infectious diseases, non-communicable diseases, LMIC

## Abstract

**Introduction:**

In sub-Saharan African settings, the increasing non-communicable disease mortality is linked to migration, which disproportionately exposes sub-populations to risk factors for co-occurring HIV and NCDs.

**Methods:**

We examined the prevalence, patterns, and factors associated with two or more concurrent diagnoses of chronic diseases (i.e., multimorbidity) among temporary within-country migrants. Employing a cross-sectional design, our study sample comprised 2144 residents and non-residents 18–40 years interviewed and with measured biomarkers in 2018 in Wave 1 of the Migrant Health Follow-up Study (MHFUS), drawn from the Agincourt Health and Demographic Surveillance System (AHDSS) in rural north-eastern South Africa. We used modified Poisson regression models to estimate the association between migration status and prevalent chronic multimorbidity conditional on age, sex, education, and healthcare utilisation.

**Results:**

Overall, 301 participants (14%; 95% CI 12.6–15.6), median age 31 years had chronic multimorbidity. Multimorbidity was more prevalent among non-migrants (14.6%; 95% CI 12.8–16.4) compared to migrants (12.8%; 95% CI 10.3–15.7). Non-migrants also had the greatest burden of dual-overlapping chronic morbidities, such as HIV-obesity 5.7%. Multimorbidity was 2.6 times as prevalent (PR 2.65. 95% CI 2.07–3.39) among women compared to men. Among migrants, men, and individuals with secondary or tertiary education manifested lower prevalence of two or more conditions.

**Discussion:**

In a rural community with colliding epidemics, we found low but significant multimorbidity driven by a trio of conditions: HIV, hypertension, and obesity. Understanding the multimorbidity burden associated with early adulthood exposures, including potential protective factors (i.e., migration coupled with education), is a critical first step towards improving secondary and tertiary prevention for chronic disease among highly mobile marginalised sub-populations.

## Introduction

In the past two post-apartheid decades, South Africa has continued to experience rising levels of within-country rural-to-urban migration, which coincides with an emerging double burden of communicable and non-communicable diseases (1). This epidemiologic transition within the epicentre of the HIV pandemic complicates population health needs as the prevalence of multimorbidity (co-occurring chronic conditions) increases and migration patterns diversify (2–4). About 25% of South African households have a member who is currently living away from home (5)—a legacy of apartheid-era policies designed to simultaneously coerce African men to move into cities to support the mining and industrial complex while prohibiting them from living in these areas permanently or bringing their rural-based families with them (6). Today, high migration flows, increasingly involving women, to rapidly urbanising locations are a constant feature associated with the search for employment opportunities and an improved quality of life (7).

Despite the urban promise of economic and education opportunities, frequent relocations disproportionately expose migrants to stressors, behaviours, occupations, and environments that put them at risk of acute and chronic health conditions (8, 9). One contributor is the “urban penalty”: migrants from rural areas often settle in crowded informal settlements near major cities where they work, and the air pollution, and sanitation challenges confer risks of respiratory illness and infectious diseases (10). These jobs are often unstable, stressful, and dangerous, placing migrants at risk of injuries and adverse mental health outcomes (11). Outside of work, living away from home uproots migrants from their social and sexual networks, which increases stress, reduces social support, and may lead to high-risk sexual encounters (12) within areas with a high prevalence of HIV. Daily life is also affected by acculturation and urbanisation: nutritional changes such as the consumption of unhealthy “fast” foods and adopting sedentary habits (11) elevate the risk of cardio-vascular outcomes. Before migrants encounter these risks, they must be healthy enough to undertake the move (13), but after migration, they often utilise healthcare services less frequently (14, 15) than their non-migrant counterparts. As a result, they may miss opportunities for disease screening, diagnoses, and treatment at a time when they need these services the most.

Examining the scale of multiple disease burden in light of health transition has been an emerging priority in South Africa with several studies linking long-term survival on antiretroviral therapy to greater risk of developing other concurrent complications (16–20). Lalkhen and Mash (21) estimated the prevalence of multimorbidity among in-patients with NCDs in South Africa. However, this study was weakened by exclusively recruiting a hospital-based population (i.e., health-seekers, likely with advanced disease), potentially underestimating the totality of patients with morbidities managed with less frequent visitations to healthcare facilities, or undiagnosed multimorbidity in communities. Only recent studies taking a population-based approach (22), have identified mechanisms behind comorbid NCDs and HIV, with prevalent NCDs being significantly associated with female gender, high cholesterol, older age, and higher wealth status (23). One study found that being an internal migrant or immigrant, was associated with lower hypertension and diabetes risk independently based on self-reported diagnoses of these morbidities (24), reflecting the varying definition and measurement considerations of migration status needed to understand the current evidence landscape.

The Agincourt Health and Demographic Surveillance System (AHDSS) *via* the Migrant Health Follow-Up Study (MHFUS), an open general population-based census and cohort set up to explore internal migration and prevalent communicable and NCDs in the Bushbuckridge District, South Africa, captures residential location data of individuals from study communities, availing a rare opportunity to cross-sectionally examine the association between internal migration (including destination factors) and multimorbidity. As research unfolds, there is greater need to broaden our understanding of the burden of multimorbidity among populations hypothesized to have an increased disease risk, such as temporary labour migrants. As opportunities for employment continue to require migration to urban settings, those who move may have poor access to preventive interventions and appropriate care for chronic conditions in the new locations. Given the high HIV risk among migrants (25–27) and associated treatment engagement complications, analysis focused on multimorbidity is a crucial first step to identify modifiable infectious and non-communicable disease risk factors, filling public health gaps.

In this study we examine prevalence, patterns, and factors associated with the multimorbidity burden within a highly mobile cohort using population-based cross-sectional data from a rural-origin community in north-eastern South Africa undergoing health transition. Building on past evidence, we hypothesized that multimorbidity is associated with a complex interplay of individual, social, and environmental factors, and is expected to vary based on age, gender, migration status, and access to healthcare.

## Methods

### Study setting

We used data from Wave 1 of the Migrant Health Follow-Up Study (MHFUS), a five-year cohort study of resident and non-resident adults aged between 18 and 40 years from the ongoing Agincourt Health and Demographic Surveillance System (AHDSS) collected in 2018 (15). The AHDSS study site, which is in the subdistrict of Bushbuckridge, Mpumalanga Province, South Africa, is characterised by high unemployment rates, such that rural livelihoods depend on labour migration. For instance, between 2013 and 2017, just over 60% of men aged 30–44 years had migrated (15) and many remit wages back to their origin households contributing to household income (28). The high prevalence of temporary migration in younger adult ages motivated the choice of age range on which the cohort was based. Analysis of prior data from the AHDSS has shown circular migration to worsen the burden of disease and mortality, demonstrated by excess AIDS- and TB-related mortality among returning migrants compared to permanent residents of the Agincourt study area between 1994 and 2006, increasing the crude death rate by 78·7% [95% CI, 77·4–80·1] for men and 44·4% [95% CI, 43·2–46·1] for women (29). However, longer duration migration among temporary migrants has been found to be associated with reduced risk of non-communicable disease mortality over time compared to non-migrants, HR = 0.60 (0.48–0.74) between 2008 and 2011 (11).

The MHFUS questionnaire collected data on residence history, socio-demographic factors and self-reported healthcare utilisation. In addition, several biomarkers were measured at baseline, Wave 1, on a sub-sample including dried-blood spot (DBS) specimens *via* a finger prick to determine HIV antibodies, and glucose. Blood glucose levels were measured through a haemoglobin A1C test. Blood pressure (BP) was measured with the Omron (HBP-1100-E), a clinically validated automatic BP monitor. Height and weight were measured, and body mass index (BMI) calculated.

### Participants and procedures

A simple random sample of 3,800 resident and non-resident adults aged between 18 and 40 years was drawn from the AHDSS, but 702 potential participants were not interviewed in Wave 1 because of ineligibility, refusals, or because they or their households were untraceable (15). Wave 1 data (2018) from the MHFUS consisted of 3,098 participants, with biomarker measures available from 2,144 people. This multimorbidity study is based upon the sub-sample of 2,144 participants who consented and had specimen collection, anthropometric measurements, and self-reported health history.

Participants were either resident (located within the surveillance site in Agincourt/Bushbuckridge) or non-resident members (i.e., migrants living outside of the study site). For recruitment of non-resident members, current contact information was availed through a member of the origin household and fieldworkers reached them in their destinations for face-to-face interviews upon scheduled visits. Although most migrants do not sever relations with the origin household (thus considered temporary migrants), MHFUS retains in its sample those individuals who are no longer considered AHDSS household members.

### Public involvement in research

The AHDSS has had a 30-year presence in the study site and established efforts to continuously involve participants through the Public Engagement Office. For instance, the political and traditional hierarchy of the constituent villages are consulted annually prior to each census launch through Agincourt’s Community Advisory Board (CAB). Importantly, focus for this study (i.e., inclusion of questions on chronic conditions and collection biological samples and data) builds on pre-existing empirical HIV and NCD mortality data in the migrant population of this rural community (8, 19, 30), serving as direct input to the design of this study. Thus, building on the long-running connection and ongoing links to the community enabled the follow-up of migrants.

### Ethics statement

Ethical approval was obtained from University of the Witwatersrand Human Research Ethics Committee (clearance certificate number M170277) and the Mpumalanga Province Human Research Ethics Committee, South Africa. Written informed consent was obtained from all participants and consent material was translated into the local Xitsonga language, with verbal consent received from those interviewed *via* telephone.

### Outcome measurement

The primary outcome of interest is multimorbidity, defined as two or more co-existing chronic (i.e., long-term) conditions, of the nine conditions that were investigated: HIV, tuberculosis (TB), hypertension, obesity, diabetes, asthma, depression, chronic obstructive pulmonary disease (COPD), and stroke.

We collected biomarker measures for four (HIV, hypertension, obesity, and diabetes) of the nine conditions. For the other five conditions, diagnoses were self-reported but not validated for all the 2,144 participants *via* either face-to-face or telephone interviews. Thus, no verification of disease status was performed through confirmatory biomarker testing or medical records at data collection for these five conditions. As per study design, BP readings were taken three times sequentially after five minutes of rest with two-minute intervals between measurements. Hypertension was defined as mean systolic BP ≥140 mm Hg or mean diastolic BP ≥90 mm Hg using the mean of the second and third BP readings, or self-reported current treatment for hypertension. Obesity was defined as BMI ≥30 kg/m^2^. Diabetes was determined by having hemoglobin A1C levels of ≥6.5% or self-reported diagnosis. In summary, 2,144 participants had both biomarker/anthropometric and self-reported data to assign disease (i.e., to merit inclusion in our multimorbidity definition), thus comprising the analytic sample for this sub-study.

#### HIV testing

In Wave 1, interview respondents were required to provide written informed consent for both completing the interview and presenting for DBS collection to enable HIV testing. DBS collection was only possible for respondents available for in-person interviews. Many respondents in more remote locations were interviewed telephonically and thus have no biomarker information; others refused DBS-HIV testing. Supplementary Table S1 presents characteristics of those included and excluded from the study. Our analytic sample of 2,144 excluded 954 participants from the original total of 3,098 due to mode of interview (phone) or refusal for anthropometric or HIV measurement. These individuals were disproportionately migrants, and particularly migrants less geographically accessible to our fieldwork teams. In turn these excluded individuals were somewhat more likely to be male, have tertiary education, and were less likely to be in the youngest (18–24) age group (see Supplementary S1). Given that migrants were more represented within the excluded population highlights challenges of collecting data for highly mobile populations, who constituted 43.1% of the wider initial 3,098 sample. For participants with complete biomarker information, positive HIV diagnosis was determined by a participant testing positive using an HIV ELISA test.

### Exposure ascertainment

The primary exposure of the study was migration status of men and women aged 18–40 years. We defined migrant status based on current residence status, defined as a respondent's current place of residence—or the place where a respondent typically spent four or more nights a week. The questionnaire validated the answer to this open-ended question through a sequence of branched questions about the province and location of current residence. Participants living in one of the 31 constituent villages of the Agincourt study site in Mpumalanga province were categorised as Agincourt residents/non-migrants, while respondents residing outside of the study area were assigned as migrants.

### Covariates

The covariates examined include sex, age, and education status. For primary healthcare system utilised, categories were western biomedical services, faith/traditional/western combined (i.e., medical pluralism) and no healthcare utilisation history in the last 12 months.

### Statistical analysis

First, we summarised participants' key socio-demographic and health characteristics by sex using descriptive statistics, frequencies, percentages and chi-squares. Second, we measured the distribution of single morbidities for participants with one condition and then repeated this by migration status, assessing the relationship between individual health conditions using correlation analysis. We further identified common dual-disease combinations and measured the frequency of disease patterns for both the migrant and non-migrant groups. Lastly, we fitted modified Poisson regression models to independently estimate the prevalence of multimorbidity for key socio-demographic and health factors. In separate models, we included interaction terms between sex, education, and migration status with multimorbidity to consider relationships suggested in prior studies (31, 32).

## Results

Of the 2,144 participants in the present multimorbidity analysis, 35% were aged between 18 and 24 years, median age 31 years (IQR = 9.4), 30% were living outside of the AHDSS study area (migrants) at the time of the Wave 1 interview, and 47% had exclusively used formal health care services in the prior year (Table 1). Using our definition of multimorbidity, 14% of respondents had at least two or more conditions. Comparing prevalence across migration profiles, 14.6% of non-migrants (95%CI, 12.8–16.4) compared to 12.9% of migrants (95% CI, 10.3–15.7) had multimorbidity. The prevalence of chronic conditions varied, with 24% (*n* = 513) being HIV positive, 19.9% (*n* = 428) having obesity, 16.6% (*n* = 356) having hypertension and 3.9% (*n* = 83) having diabetes. There were significant differences in the prevalence of conditions by migration status, 25.3% of non-migrants compared to 20.7% migrants had tested HIV positive, 20.5% non-migrants vs. 18.7% migrants had obesity and more migrants (21.9%) compared to non-migrants (14.4%) had hypertension. We detected non-negligible differences by migration status in the distributions for gender, age, education status, primary healthcare system, number of health conditions and the prevalence of “Other” (low prevalence) conditions.

**Table 1:** Description of the sociodemographic and health characteristics of adults in the multimorbidity sample from the migrant health follow-up study (MHFUS) (*N* = 2,144).

	Overall	Migrant	Non-migrant	Statistics
	*N* (%)	*n* (%)	*n* (%)	
**Migration status**
Migrant	632 (29.5)			
Non-migrant	1,512 (60.5)			
**Sex**
Female	1,131 (52.7)	282 (44.6)	849 (56.2)	*χ2 (1) = 203.11, p < 0.01*
Male	1,013 (47.3)	350 (55.4)	663 (43.8)	
**Age categories**
18–24 years	744 (34.7)	164 (29.9)	580 (38.4)	*χ2 (3) = 203.11, p < 0.01*
25–29 years	566 (26.4)	217 (34.4)	349 (23.1)	
30–34 years	485 (22.6)	151 (23.9)	334 (22.1)	
≥35 years	349 (16.3)	100 (15.8)	249 (16.5)	
**Highest education**
Primary	95 (4.4)	15 (2.4)	80 (5.3)	*χ2 (2) = 38.24, p < 0.01*
Secondary	1,724 (80.4)	477 (75.3)	1,247 (82.5)	
Tertiary	325 (15.2)	140 (22.2.)	185 (12.2)	
**Primary Healthcare system**
Western biomedical	998 (46.6)	190 (30.1)	808 (53.4)	*χ2 (2) = 115,77, p < 0.01*
Faith/Traditional/Western	107 (5.0)	62 (9.8)	45 (3.0)	
No healthcare utilization	1,039 (48.4)	380 (60.1)	659 (44.6)	
**No. of conditions**
0	1,089 (50.8)	315 (49.8)	774 (51.2)	*χ2 (1) = 96.62, p < 0.01*
1	754 (35.2)	236 (37.3)	518 (34.3)	
2+	301 (14.0)	81 (12.9)	220 (14.6)	
**Health condition**
HIV	513 (23.9)	131 (20.7)	382 (25.3)	*χ2 (1) = 5.04, p < 0.05*
Hypertension	356 (16.6)	139 (21.9)	217 (14.4)	*χ2 (1) = 18.80, p < 0.01*
Diabetes	83 (3.9)	25 (3.9)	58 (3.8)	*χ2 (1) = 0.02, p = 0.90*
Obesity	428 (19.9)	118 (18.7)	310 (20.5)	*χ2 (1) = 0.94, p = 0.33*
Other^a^	31 (1.4)	2 (1.9)	29 (0.3)	*χ2 (1) = 8.02, p < 0.01*

^a^ Other conditions include Chronic Obstructive Pulmonary Disease (COPD), Asthma, Depression, Stroke and Tuberculosis which were established through self-reports while HIV, Hypertension, Diabetes and Obesity were biomarker validated representing those consenting to sample collection or anthropometric measurement.

**Figure 1:**
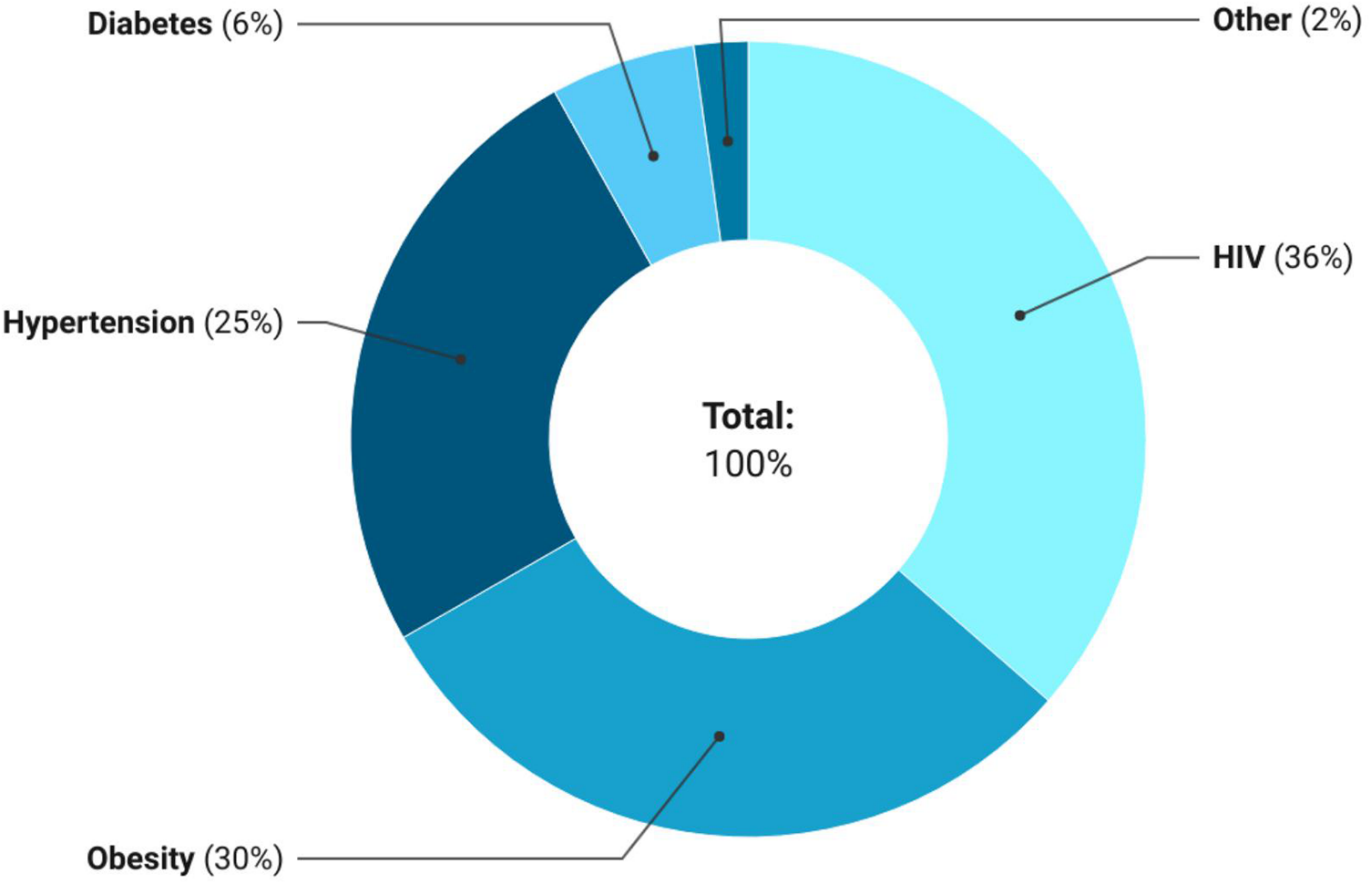
Distribution of existing single diseases among those with at least one morbidity in the MHFUS (*n* = 1,055).

A total of 1,055 adults had at least one of nine conditions (Figure 1). HIV accounted for the largest share of single disease morbidities 36% (95% CI, 33.8–38.9) followed by obesity 30.3% (95% CI, 27.9–32.8) and hypertension 25.2% (95% CI, 22.9–27.6).

Table 2 presents the correlation matrix of pairwise comorbidities for those with at least one condition i.e., 1,055 of 2,144 participants. To highlight significant results, the largest correlation between health conditions was between hypertension and obesity (tetrachoric rho = 0.31). We also observed large correlations between diabetes and obesity (tetrachoric rho = 0.24) as well as HIV and other lesser represented chronic conditions (tetrachoric rho = 0.26). This reflects the clinical importance of both obesity and HIV, as other additional health conditions (hypertension and diabetes) were associated with being obese or HIV positive.

**Table 2:** Correlation matrices for pairwise comorbidities in the MHFUS for those with two or more conditions (*N* = 301).

	Hypertension	HIV	Diabetes	Obesity	Other
Hypertension	1.00				
HIV	−0.08	1.00			
Diabetes	0.05		1.00		
Obesity	0.31^a^	0.11^a^	0.24^a^	1.00	
Other	−0.20	0.26^a^	−1.00	0.01	1.00

Conditions classified as “other” include Chronic Obstructive Pulmonary Disease (COPD), Asthma, Depression, Stroke and Tuberculosis (TB). Tetrachoric eigenvalues with an asterisk “*” indicated high positive correlation.

Correlation codes.

**Table:** 

	1.0 Perfect correlation
	0.4 High
	0.2 Intermediate
	0.1 Low
	−1.0 Negative

We found that hypertension and obesity comorbidity accounted for 3.9% (95% CI, 3.1–4.8) of all those with two or more diseases (*n* = 84 out of 2144) (Table 3). Non-migrants compared to migrants had a greater burden of dual overlapping chronic and cardio-metabolic conditions, such as HIV-obesity (4%) *p* < 0.001 and diabetes-obesity pairings (1%) *p* < 0.001. In other infrequent combinations, obesity co-occurred with several other respiratory and metabolic disorders such as TB and diabetes.

**Table 3:** Dual-disease combinations by migration status .

	HIV Hypertension		HIV Obesity		Hypertension Obesity		Diabetes Obesity		Diabetes Hypertension	
Migrant (*n* = 632)	24 (3.7%)	*p* < 0.001	26 (2%)	*p* = 0 < 001	26 (3.9%)	*p* < 0.001	12 (0.9%)	*p* < 0.001	6 (0.4%)	*p* < 0.001
Non-migrant (*n* = 1,512)	15 (1.0%)		97 (5.5%)		58 (3.8%)		19 (1.1%)		10 (0.6%)	
*N* = 2,144 (100%)	39 (1.8%)	123 (5.7%)	84 (3.9%)	31 (1%)	16 (0.5%)

*p*-values based on chi-squares of each disease pairings and migration status dichotomy.

### Factors associated with multimorbidity

Several sociodemographic factors (age, sex, education, and healthcare utilisation) were associated with multimorbidity in multivariate regressions (Table 4). Multimorbidity was higher in women compared to men, those aged 25–29 years, 30–34 years and ≥35 years compared to 18–24 years, individuals using formal healthcare exclusively in the past 12 months compared to those with no such healthcare use history. In contrast, multimorbidity was lower among individuals with secondary education (PR = 0.57, 95% CI, 0.39–0.84) compared to primary.

**Table 4:** Multimorbidity prevalence ratios for socio-demographic and health factors in a rural population, South Africa (*N* = 2,144).

	Category	Crude PR	Robust (SE)	(95% CI)
Sex: (Men)	Women	**2.65**	**0.33**	**2.07–3.39**
Age categories: (18–24 years)	25–29 years	**2.32**	**0.45**	**1.58–3.40**
	30–34 years	**3.88**	**0.71**	**2.71–5.54**
	≥35 years	**5.61**	**1.01**	**3.95–7.97**
Highest education: (Primary)	Secondary	**0.57**	**0.11**	**0.39–0.84**
	Tertiary	0.68	0.15	0.43–1.06
Primary healthcare system: (None)	Western biomedical	**1.96**	**0.23**	**1.56–2.45**
	Faith/Traditional/Western	1.26	0.35	0.73–2.17
Migration status: (non-migrant)	Migrant	0.88	0.11	0.69–1.12
Interaction terms of sex and migration status: (Women and non-migrant)	Women and migrant	1.00	0.14	0.76–1.31
	Men and non-migrant	**0.39**	**0.06**	**0.29–0.52**
	Men and migrant	**0.36**	**0.07**	**0.24–0.54**
Interaction terms for education and migration status: (Primary and non-migrant)	Primary and migrant	1.19	0.56	0.47–3.01
	Secondary and non-migrant	**0.57**	**0.13**	**0.37–0.88**
	Secondary and migrant	**0.62**	**0.15**	**0.39–0.99**
	Tertiary and non-migrant	0.98	0.25	0.60–1.61
	Tertiary and migrant	**0.32**	**0.12**	**0.15–0.65**

[Reference category in brackets].

PR, prevalence ratio; CI, confidence intervals; SE, standard error.

Table 4 highlights further results based on interaction effects. Prevalent multimorbidity was low among both migrant and non-migrant men compared to non-migrant women, with a prevalence ratio of (PR = 0.36, 95% CI, 0.24–0.54) and (PR = 0.39, 95% CI, 0.29–0.52), respectively, however migrant-non-migrant differences among men were negligible. While greater educational attainment is associated with lower prevalence of multimorbidity, as above, further differentiation by migrant status is modest. Migrant-non-migrant differences among those with secondary education are marginal (PR = 0.62, 95% CI, 0.39–0.99 vs. PR = 0.57 (95% CI, 0.37–0.88). Migrant-non-migrant differences among those with tertiary education are more evident (PR = 0.32, 95% CI, 0.15–0.65 vs. PR = 0.98, CI, 0.60–1.61).

## Discussion

This study set out to characterise multimorbidity patterns and identify associated risk factors, including migration status, in a rural community in north-eastern South Africa. In this study sample of persons aged 18–40, we found that 14% of the participants had prevalent chronic multimorbidity (≥2 conditions). Migrants exhibited a slightly lower prevalence of multimorbidity than non-migrants. HIV (24%) was the most prevalent morbidity; for instance, 25.3% of non-migrants and 20.7% of migrants had tested HIV positive. Overall, obesity and hypertension had greater co-existence in the prevalence of pairwise morbidities of the four measured conditions and five other minor conditions. The prevalence ratio for multimorbidity 2.65 higher among women compared to men and 5.61 higher among those aged over 35 years compared to 18–24 years.

HIV, hypertension and obesity were somewhat equally represented among the nine conditions considered in this study. This disease profile mirrors a turning point through early phase health transition marked by waning dominance of infectious diseases before experiencing an era of chronic non-communicable diseases (NCDs) (33). This may be in large part due the adoption of unhealthy, sedentary habits that include reduced exercise, poor dietary choices, alcohol consumption and smoking (34) Rural communities represent an important epidemiological paradox; they serve as a net receiver of return-migrants who potentially have higher levels of exposure to HIV or NCD risk factors (8, 35, 36) than the permanent residents of the rural “home”. Health facilities in rural areas face an increasing demand for appropriate care for chronic conditions in the context of underdeveloped secondary care models (20).

The attenuating effect of higher education status on multimorbidity for migrants provides important insight into identifying protective characteristics against adverse consequences in the context of ongoing temporary migration in South Africa. Mobility increases the burden of morbidity by disconnecting patients from clinic-based HIV care (i.e., leading to poor drug adherence) and erratic secondary healthcare utilisation, which delays disease prevention, diagnoses and treatment normally supported through routine NCD screening and continued engagement to care (37, 38). Recent evidence from the Agincourt study area showed that migrants compared to non-migrants, and men compared to women, each had ∼70% lower odds of health service use in the last 12-months among individuals living with a chronic condition (15). However to interpret of our finding, there are two scenarios to consider; that educational and health selectivity upon migration (39, 40) or observed health and wellbeing benefits occurring post-migration (41) is mediated through improved socio-economic status (42, 43), which cannot be demonstrated using a cross-sectional design. Taking previous data into consideration, we posit that while migrants are at risk of gaps in healthcare engagement continuity, being more educated (i.e., higher socio-economic status and health literacy) helps overcoming barriers to accessing healthcare (i.e., both when at destination and origin) confers the preventive benefit to multimorbidity. Despite concerns about potential misclassification, we provide important single time-point data to support the health advantage enjoyed by more educated migrants from rural areas residing in urban areas, thus helping to close the health gap across by migration status.

Our study further demonstrates lower prevalent multimorbidity among migrant men however, migrant women registered no such significantly lower rates which may be due to gendered destination dynamics systematically promoting men and not women's health. Beyond the stressful migration process, women are expected to continue social roles such as being the homemaker and taking care of family and may lack opportunities for social integration compared to men (44). We echo others' (45) attribution of elevated BP measures, a driver of syndemic NCDs—diabetes and obesity, among migrant women compared to men. Thus, indicative of two scenarios; that women either accrue fewer health benefits from urban living than men do, or the benefits women accrue do not manifest as lower BP as is the case for men (45).

The interpretation of our results should be in view of several limitations. We examined migration status (residing inside or outside the rural-origin Agincourt HDSS area) at a point in time using data from the first study wave. Thus, we do not account for prior years' migration history in the analysis. Moreover, the cross-sectional approach limits our ability to examine the timing of migration, diagnosis of HIV and co-existing conditions. More recently evidence exists that the migrant health advantage recedes over time, even for within-country migrants (44) warranting the need for more research accounting for other migration dimensions such as duration and recency. A strength of our study is being the first in South Africa to analyse population specific survey data recording residential patterns, sampling migrants from a rural origin area and capturing destination circumstances, such as healthcare utilisation behaviours while away from home. Community health screening data such as contained in the MHFUS provide unique non-clinic-based estimates of undiagnosed multimorbidities thus using a more versatile design than previous studies (46).

Understanding the heightened multimorbidity burden that occurs with migration is an essential first step towards improving preventive interventions for marginalised populations and underserved communities including spatially optimised access to appropriate care for mobile individuals. Results from this study address clinical and public health gaps, implying the need for expanded scope of community-based health screening services beyond HIV to include NCDs and the risk factors preceding morbidity (22). In keeping with this, classification of the four main morbidities examined in this research study, HIV, hypertension, and obesity were ascertained through biomarker data collection, which can be a model for the expansion of testing services into communities, which is a new focus in South Africa (47). Building on learnings from HIV care services (with a comprehensive network of intermediaries in South Africa), healthcare delivery can also be adapted to monitor comorbidities and NCDs in young women in particular (48). Additionally, interventions involving early initiation of longer-term life-course care can be explored, building on debut presentation of women for sexual reproductive health services (49, 50). Awareness interventions for younger populations can be uniquely customised to counteract personal perceptions (51–53). For instance, these may help to improve the effectiveness of weight loss campaigns and help in tackling obesity.

## Conclusions

In a rural South African community with colliding epidemics of communicable and non-communicable diseases, we assessed multimorbidity prevalence. Among adults aged 18–40, we found high prevalent trio conditions HIV, obesity, and hypertension. Migrants were observed to have multimorbidity pairings that were higher for HIV-Hypertension and lower for HIV-Obesity. Unadjusted migrant-non-migrant differentials in overall multimorbidity prevalence were modest, but selected interactions with demographic characteristics were observed in multivariate statistical analysis. These multivariate statistical analyses also revealed, notably, that women, those with limited education, and those older than 24 years manifested a higher prevalence of two or more conditions. These data highlight longstanding gaps in achieving health equity for these vulnerable groups. Understanding the potential role of education in disrupting the multimorbidity patterns associated with early-life exposures such as migration is a critical first step towards improving chronic disease preventive interventions for marginalised populations and underserved communities including spatial optimisation of access to secondary care for those mobile.

## Data Availability

Data from the Migrant Health Follow-Up Study are available from the corresponding author on valid request. Agincourt Health and Demographic Surveillance Systems data are available through SAPRIN URL http://saprin.mrc.ac.za/ and http://saprindata.samrc.ac.za/.
