# Supplementary material for: Epidemiology of chronic multimorbidity and temporary migration in a rural South African community in health transition: A cross-sectional population-based analysis
Source: Front Epidemiol. 2023 Mar 21;3:1054108. doi: 10.3389/fepid.2023.1054108 (PMC10910947; doi:10.3389/fepid.2023.1054108)
Supplement: Supplementary file 1 [file Datasheet1.pdf]

Table S1. Comparison of sociodemographic and health characteristics of MHFUS cohort participants in the multimorbidity study subsample (N=2144) and those excluded (N= 954)

	Multimorbidity subsample of MHFUS N=2144		Not in MHFUS multimorbidity subsample, N=954		p-values of contrasts
	N	%	n	%	
Migration status					
Migrant	632	(29.5)	702	(73.6)	p<0.001
Non-migrant	1 512	(60.5)	252	(26.4)	
Sex					
Male	1 131	(52.8)	545	(57.1)	p<0.001
Female	1 013	(47.2)	409	(42.9)	
Age categories					
18-24y	744	(34.7)	191	(20.0)	p<0.001
25-29y	566	(26.4)	292	(30.6)	
30-34y	485	(22.6)	255	(26.7)	
≥35y	349	(16.3)	216	(22.6)	
Highest education					
Primary	95	(4.4)	19	(2.0)	p<0.001
Secondary	1 724	(80.4)	738	(77.4)	
Tertiary	325	(15.2)	197	(20.6)	
Primary Healthcare system					
Western biomedical	998	(46.6)	378	(39.6)	p<0.001
Faith/Traditional/Western	107	(5.0)	37	(3.8)	
No healthcare utilization	1 039	(48.4)	539	(56.5)	
Health conditions from Questionnaire					
COPD	1	(0.1)	0	(0.0)	p=0.505
Asthma	16	(0.8)	3	(0.3)	p=0.155
Depression	5	(0.3)	1	(0.1)	p=0.453
Stroke	1	(0.1)	0	(0.0)	p=0.505
TB	8	(0.4)	2	(0.2)	p=0.459
No. of above conditions					
0	2 113	(98.6)	948	(99.4)	p<0.001
1	31	(1.4)	6	(0.6)	
2	0	(0.0)	0	(0.0)	
3+	0	(0.0)	0	(0.0)	
